# Supplementary material for: Heparin-based hydrogel scaffolding alters the transcriptomic profile and increases the chemoresistance of MDA-MB-231 triple-negative breast cancer cells
Source: Biomater Sci. 2020 Feb 13;8(10):2786–96. doi: 10.1039/c9bm01481k (PMC7497406; doi:10.1039/c9bm01481k)
Supplement: Supplementary file 2 [file BM-008-C9BM01481K-s002.zip › Supplementary File 4/EGFvControl/Pathways/my_analysis.Gsea.1545200981068/gsea_report_for_CONTROL_1545200981068.html]

Report for CONTROL 1545200981068 [GSEA]

| GS  follow link to MSigDB | GS DETAILS | SIZE | ES | NES | NOM p-val | FDR q-val | FWER p-val | RANK AT MAX | LEADING EDGE || 1 | HALLMARK\_COAGULATION | Details ... | 87 | -0.60 | -2.61 | 0.000 | 0.000 | 0.000 | 2504 | tags=46%, list=13%, signal=53% |
| 2 | HALLMARK\_HYPOXIA | Details ... | 163 | -0.54 | -2.60 | 0.000 | 0.000 | 0.000 | 3585 | tags=53%, list=19%, signal=65% |
| 3 | HALLMARK\_TNFA\_SIGNALING\_VIA\_NFKB | Details ... | 178 | -0.52 | -2.49 | 0.000 | 0.000 | 0.000 | 3604 | tags=53%, list=19%, signal=65% |
| 4 | HALLMARK\_KRAS\_SIGNALING\_UP | Details ... | 144 | -0.48 | -2.24 | 0.000 | 0.000 | 0.000 | 2392 | tags=35%, list=12%, signal=39% |
| 5 | HALLMARK\_INTERFERON\_ALPHA\_RESPONSE | Details ... | 91 | -0.50 | -2.23 | 0.000 | 0.000 | 0.001 | 3408 | tags=43%, list=18%, signal=52% |
| 6 | HALLMARK\_EPITHELIAL\_MESENCHYMAL\_TRANSITION | Details ... | 150 | -0.47 | -2.22 | 0.000 | 0.000 | 0.001 | 2873 | tags=40%, list=15%, signal=47% |
| 7 | HALLMARK\_INTERFERON\_GAMMA\_RESPONSE | Details ... | 167 | -0.44 | -2.12 | 0.000 | 0.000 | 0.001 | 3408 | tags=41%, list=18%, signal=49% |
| 8 | HALLMARK\_IL6\_JAK\_STAT3\_SIGNALING | Details ... | 63 | -0.51 | -2.08 | 0.000 | 0.000 | 0.001 | 3598 | tags=49%, list=19%, signal=60% |
| 9 | HALLMARK\_INFLAMMATORY\_RESPONSE | Details ... | 130 | -0.43 | -2.00 | 0.000 | 0.000 | 0.002 | 3924 | tags=45%, list=20%, signal=57% |
| 10 | HALLMARK\_COMPLEMENT | Details ... | 141 | -0.42 | -1.97 | 0.000 | 0.000 | 0.002 | 2834 | tags=33%, list=15%, signal=39% |
| 11 | HALLMARK\_IL2\_STAT5\_SIGNALING | Details ... | 155 | -0.41 | -1.95 | 0.000 | 0.000 | 0.003 | 2803 | tags=35%, list=15%, signal=41% |
| 12 | HALLMARK\_CHOLESTEROL\_HOMEOSTASIS | Details ... | 67 | -0.44 | -1.82 | 0.000 | 0.002 | 0.011 | 3604 | tags=40%, list=19%, signal=49% |
| 13 | HALLMARK\_MYOGENESIS | Details ... | 135 | -0.39 | -1.77 | 0.000 | 0.003 | 0.020 | 3259 | tags=37%, list=17%, signal=44% |
| 14 | HALLMARK\_APOPTOSIS | Details ... | 140 | -0.37 | -1.77 | 0.000 | 0.003 | 0.021 | 3106 | tags=34%, list=16%, signal=41% |
| 15 | HALLMARK\_APICAL\_JUNCTION | Details ... | 147 | -0.37 | -1.73 | 0.000 | 0.004 | 0.032 | 2358 | tags=27%, list=12%, signal=31% |
| 16 | HALLMARK\_ANGIOGENESIS | Details ... | 23 | -0.53 | -1.70 | 0.016 | 0.005 | 0.045 | 2504 | tags=48%, list=13%, signal=55% |
| 17 | HALLMARK\_ESTROGEN\_RESPONSE\_LATE | Details ... | 161 | -0.34 | -1.63 | 0.003 | 0.008 | 0.082 | 2119 | tags=27%, list=11%, signal=30% |
| 18 | HALLMARK\_ALLOGRAFT\_REJECTION | Details ... | 116 | -0.35 | -1.60 | 0.000 | 0.010 | 0.103 | 2069 | tags=28%, list=11%, signal=31% |
| 19 | HALLMARK\_GLYCOLYSIS | Details ... | 171 | -0.32 | -1.55 | 0.000 | 0.014 | 0.154 | 2269 | tags=24%, list=12%, signal=27% |
| 20 | HALLMARK\_BILE\_ACID\_METABOLISM | Details ... | 80 | -0.35 | -1.51 | 0.017 | 0.018 | 0.191 | 2021 | tags=23%, list=11%, signal=25% |
| 21 | HALLMARK\_KRAS\_SIGNALING\_DN | Details ... | 87 | -0.35 | -1.51 | 0.015 | 0.018 | 0.206 | 3114 | tags=33%, list=16%, signal=40% |
| 22 | HALLMARK\_ESTROGEN\_RESPONSE\_EARLY | Details ... | 170 | -0.31 | -1.51 | 0.003 | 0.018 | 0.206 | 3104 | tags=30%, list=16%, signal=35% |
| 23 | HALLMARK\_P53\_PATHWAY | Details ... | 179 | -0.31 | -1.48 | 0.003 | 0.022 | 0.265 | 3250 | tags=30%, list=17%, signal=35% |
| 24 | HALLMARK\_HEDGEHOG\_SIGNALING | Details ... | 26 | -0.41 | -1.38 | 0.094 | 0.044 | 0.481 | 3338 | tags=42%, list=17%, signal=51% |
| 25 | HALLMARK\_HEME\_METABOLISM | Details ... | 166 | -0.26 | -1.24 | 0.049 | 0.125 | 0.859 | 2979 | tags=24%, list=16%, signal=28% |
| 26 | HALLMARK\_TGF\_BETA\_SIGNALING | Details ... | 50 | -0.32 | -1.24 | 0.143 | 0.123 | 0.865 | 1701 | tags=18%, list=9%, signal=20% |
| 27 | HALLMARK\_ANDROGEN\_RESPONSE | Details ... | 91 | -0.25 | -1.11 | 0.254 | 0.284 | 0.994 | 1977 | tags=19%, list=10%, signal=21% |
| 28 | HALLMARK\_NOTCH\_SIGNALING | Details ... | 28 | -0.32 | -1.09 | 0.323 | 0.315 | 0.996 | 2267 | tags=21%, list=12%, signal=24% |
| 29 | HALLMARK\_APICAL\_SURFACE | Details ... | 29 | -0.32 | -1.08 | 0.321 | 0.315 | 0.997 | 3559 | tags=34%, list=19%, signal=42% |
| 30 | HALLMARK\_XENOBIOTIC\_METABOLISM | Details ... | 151 | -0.20 | -0.94 | 0.582 | 0.624 | 1.000 | 2030 | tags=15%, list=11%, signal=17% |
| 31 | HALLMARK\_PEROXISOME | Details ... | 85 | -0.20 | -0.88 | 0.704 | 0.776 | 1.000 | 2021 | tags=15%, list=11%, signal=17% |
| 32 | HALLMARK\_WNT\_BETA\_CATENIN\_SIGNALING | Details ... | 35 | -0.22 | -0.80 | 0.779 | 0.892 | 1.000 | 3909 | tags=34%, list=20%, signal=43% |
Table: Gene sets enriched in phenotype **CONTROL (2 samples) **[plain text format]****
